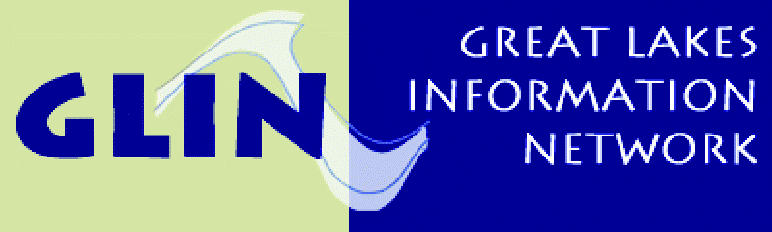# EHPnet: Great Lakes Information Network

**Published:** 2005-03

**Authors:** Erin E. Dooley

The Great Lakes Information Network (GLIN) is a partnership of public and private organizations managed by the Great Lakes Commission, an eight-state compact agency. The GLIN website, located at **http://www.great-lakes.net/**, serves as a central online source for information about the Great Lakes region. A strong network of state, provincial, federal, and regional partner agencies and organizations populate the GLIN with essential information for use by policy makers and others with an interest in the region.

The site’s homepage is divided into six different topic sections, plus a daily news page featuring current news articles, press releases, announcements, and links to information about lake conditions and weather forecasts. Visitors can sign up to receive daily news or to submit stories.

The Great Lakes section of the site features an interactive map of the region. Clicking on different geographic areas brings up state-, province-, or lake-specific news as well as links to general resources (such as governmental programs, state facts, and information on Sea Grant programs), economic profiles, environmental information, and tourism sites.

The Environment section of the GLIN site groups information into five topics, each with several subtopics. The main topics are Air and Land, Water, Flora and Fauna, Pollution, and References. Subtopics include sustainable development, air toxics, human health, toxic contamination, and environmental justice, among many others. This section also provides an overview of the Great Lakes natural environment and general resources including an online atlas of the region published by the U.S. Environmental Protection Agency, back and current issues of Environment Canada’s *Science and the Environment Bulletin*, and the Commission for Environmental Cooperation’s summary of environmental law in North America.

Similarly, the Economy section includes information grouped under the main topics of Business Development, Business and Environment, and References, with agriculture, energy, economic development, and transportation among the subtopics.

Aimed specifically for teachers, the Education portion of the site features free online “mini-lesson” modules for students in elementary through high school. Subjects covered in the lessons include the natural environment, geography, and pollution. The natural environment module features information on water diversion out of the region, invasive species, and protection of water quality. The causes and effects of Great Lakes pollution is the main focus of the pollution unit, which also includes a section on urban sprawl. An online glossary helps students with unfamiliar terms used throughout the modules.

The Maps and GIS (geographic information systems) section of the site offers resources to help visitors customize their own Great Lakes map using GIS data (searchable by topic, region, or organization), online mapping tools, and a gallery of free downloadable maps. The types of maps offered range from ecological assessment maps to satellite images.

Finally, the Tourism section of the site, with its extensive list of local recreational, cultural, and historical offerings, provides one small hint of the richness of this magnificent natural resource we call the Great Lakes.

## Figures and Tables

**Figure:**